# Expression of Interleukin 6 signaling receptors in carotid atherosclerosis

**DOI:** 10.1177/1358863X20977662

**Published:** 2020-12-22

**Authors:** Louise Ziegler, Jasmin Lundqvist, Kristian Dreij, Håkan Wallén, Ulf de Faire, Gabrielle Paulsson-Berne, Ulf Hedin, Ljubica Matic, Bruna Gigante

**Affiliations:** 1 Department of Clinical Sciences, Danderyd Hospital, Karolinska Institutet, Stockholm, Sweden; 2 Department of Medicine, Danderyd Hospital, Stockholm, Sweden; 3 Cardiovascular Medicine Unit, Department of Medicine, Karolinska Institutet, Stockholm, Sweden; 4 Unit of Biochemical Toxicology, Institute of Environmental Medicine, Karolinska Institutet, Stockholm, Sweden; 5 Department of Cardiology, Danderyd Hospital, Stockholm, Sweden; 6 Unit of Cardiovascular and Nutritional Epidemiology, Institute of Environmental Medicine, Karolinska Institutet, Stockholm, Sweden; 7 Department of Cardiology, Karolinska University Hospital, Stockholm, Sweden; 8 Department of Vascular Surgery, Karolinska University Hospital, Stockholm, Sweden; 9 Department of Molecular Medicine and Surgery, Karolinska Institutet, Stockholm, Sweden

**Keywords:** atherosclerosis, carotid artery disease, cerebrovascular disease, inflammation, interleukin 6 (IL6)

## Abstract

Interleukin (IL) 6 contributes to atherosclerotic plaque development through IL6 membrane-bound (IL6R and gp130) and soluble (sIL6R and sgp130) receptors. We investigated IL6 receptor expression in carotid plaques and its correlation with circulating IL6 and soluble receptor levels. Plasma samples and carotid plaques were obtained from 78 patients in the Biobank of Karolinska Endarterectomies study. IL6, sIL6R, and sgp130 were measured in plasma and *IL6, IL6R, sIL6R, GP130*, and s*GP130-RAPS* (s*GP130*) gene expression assessed in carotid plaques. Correlations between plaque IL6 signaling gene expression and plasma levels were determined by Spearman’s correlation. Differences in plasma and gene expression levels between patients with (*n* = 53) and without (*n* = 25) a history of a cerebral event and statin-treated (*n* = 65) and non-treated (*n* = 11), were estimated by Kruskal–Wallis. *IL6* and its receptors were all expressed in carotid plaques. There was a positive, borderline significant, moderate correlation between plasma IL6 and sIL6R and the respective gene expression levels (rho 0.23 and 0.22, both *p* = 0.05). *IL6R* expression was higher in patients with a history of a cerebrovascular event compared to those without (*p* = 0.007). Statin-treated had higher *IL6R, sIL6R*, and s*GP130* expression levels and plasma sIL6R compared to non-treated patients (all *p* < 0.05). In conclusion, all components of the IL6 signaling pathways are expressed in carotid artery plaques and IL6 and sIL6R plasma levels correlate moderately with *IL6* and *sIL6R*. Our data suggest that IL6 signaling in the circulation might mirror the system activity in the plaque, thus adding novel perspectives to the role of IL6 signaling in atherosclerosis.

## Introduction

Inflammation is the hotbed for atherosclerosis and the pro-inflammatory interleukin (IL) 1β-IL6-C-reactive protein (CRP) pathway is the central axis. In the midst of the pathway, IL6 is a key player associated with carotid atherosclerotic plaques.^1–3^

IL6 governs pivotal physiological processes (e.g. immunological and regenerative) through the binding to the membrane-bound IL6 receptor (IL6R), a signaling moiety known as classical signaling. On the other side, IL6 elicits pro-inflammatory signals in target tissues when bound to the soluble isoform of IL6R (sIL6R),^4^ in the so-called IL6 trans-signaling. Both signaling moieties need the ubiquitously expressed co-receptor gp130 to transduce the IL6 signal. To avoid systemic uncontrolled inflammation, the circulating IL6:sIL6R complex is buffered by a soluble gp130 isoform (sgp130).^5^ The sIL6R and sgp130 are produced either through limited proteolytic cleavage of the membrane-bound receptor (main mechanism for sIL6R) or by alternative splicing of the gene coding the transmembrane receptor (main mechanism for sgp130).^6,7^

We previously demonstrated that IL6 trans-signaling, estimated by high values of the ratio between the circulating active IL6:sIL6R and inactive IL6:sIL6R:sgp130 complex, was associated with an increased risk of future cardiovascular events (CVE)^8^ and early ischemic stroke in middle-aged individuals without prevalent cardiovascular disease and low-normal low-density lipoprotein (LDL)-cholesterol levels.^9^ The association of IL6 and IL6 trans-signaling with CVE risk has, however, thus far solely been analyzed in relation to peripheral circulating levels and, apart from previous studies on the vascular expression of *IL6*, gene expression of the membrane-bound and soluble receptors in the IL6 pathways has not been investigated in atherosclerotic plaques in vivo.^10^ Hence, analyzing the IL6 receptor gene expression in human atherosclerotic plaques might provide novel insights to target inflammation in atherosclerosis. Of interest, targeting IL6 trans-signaling with recombinant sgp130 (sgp130Fc) attenuates atherosclerotic plaques in mice.^11^

The primary aim of the present study was to investigate if *IL6* and the genes encoding its membrane-bound and soluble receptors are expressed in carotid atherosclerotic plaques from patients undergoing carotid endarterectomy (CEA). The secondary aim was to investigate potential correlations between plaque gene expression levels and plasma levels of IL6 and its soluble receptors. In exploratory analyses, we describe the gene expression and plasma levels in clinically relevant subgroups.

## Methods

### Study population

**Figure 1.**
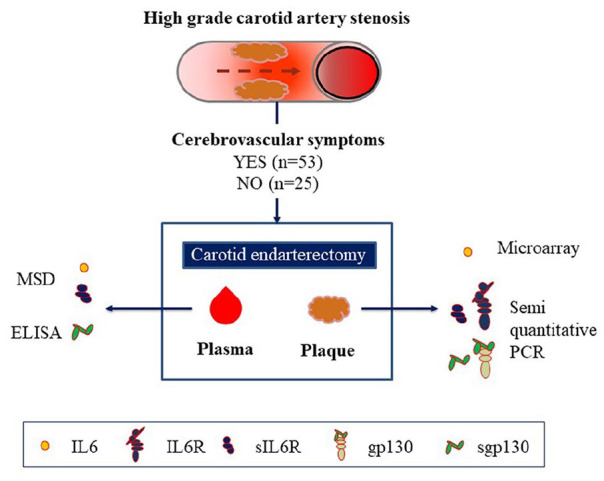
Study population and methods. ELISA, enzyme-linked immunosorbent assay; gp130, glycoprotein 130; IL6, interleukin 6; IL6R, IL6 receptor; MSD, Meso Scale Discovery Systems; PCR, polymerase chain reaction; sgp130, soluble glycoprotein 130; sIL6R, soluble IL6 receptor.

Patients with high-grade carotid artery stenosis, according to the North American Symptomatic Carotid Endarterectomy Trial (NASCET) criteria,^12^ who were amenable for CEA in Stockholm County in 2002–2007 were consecutively offered to be included in the Biobank of Karolinska Endarterectomies (BiKE) study.^13^ Participants with a history of transient (< 24 hours) cerebral symptoms or who had suffered from permanent neurological deficit secondary to an ischemic insult in the arteria media territory contralateral to the stenosed carotid artery < 6 months prior to CEA were categorized as symptomatic and otherwise as asymptomatic (i.e. no cerebral symptoms or symptoms ⩾ 6 months before CEA). Patients with extensive deficit and large cerebral infarcts and patients with atrial fibrillation were not included in the study. Epidemiological data, clinical blood measurements, history of diseases and medication and anthropometric data were collected. CEA was performed with local anesthesia. A schematic representation summarizing the study population and the methods of the study is presented in Figure 1.

The current analysis was performed in a subset of the BiKE cohort, the so-called ‘discovery’ dataset, with patients consecutively recruited between 2005 and 2009 (*n* = 97).^10^ No clinical selection was applied for this subset other than the availability and good quality of the RNA isolated from plaques as per the technical standards needed for analyses.

Owing to incomplete clinical data (*n* = 4), a SD > 0.5 in the semi-quantitative real-time polymerase chain reaction (semi-qRT-PCR) (*n* = 4) or lacking adequate semi-qRT-PCR samples (*n* = 11), 19 subjects were excluded from analysis. Among the remaining 78 patients included in the present analysis, 53 were classified as symptomatic and 25 were classified as asymptomatic.

The BiKE study was approved by the Ethical Committee of North Stockholm with the following ethical permit numbers: 95-276/277; 01-199; 02-147; 2009/512-31/2. The study followed the guidelines of the 1964 Declaration of Helsinki. All human samples and data in the BiKE study were collected with informed consent from patients.

### Biochemical analysis of blood samples

Blood samples drawn at surgery were instantly processed to plasma, which was then aliquoted and frozen to –80°C. The study protocol for the blood sample collection in BiKE has been reported before.^14^ Each one of the three biomarkers, IL6, sIL6R, and sgp130, was measured in all study participants at the same time. IL6 (pg/mL) and sIL6R (ng/mL) were measured by Meso Scale Discovery cytokine assay (Gaithersburg, MD, USA). Plasma levels of sgp130 were assessed using a developmental kit (#DY228) from R&D Systems (Minneapolis, MN, USA).^8^ All analyses were performed in accordance with the manufacturer’s assay protocol.

### Analysis of the expression of IL6 signaling components in plaques

In each patient, the carotid atherosclerotic plaque retrieved were cut in half at the most stenotic part and the entire proximal portion was immediately frozen at −80°C. The halved plaque was later homogenized and total RNA was extracted from the homogenate and reverse transcribed to cDNA as previously described.^15^ *IL6* expression (log2) was estimated by GeneChip™ Human Genome U133 Plus 2.0 Array (Thermo Fisher Scientific, Santa Clara, CA) as reported in a prior publication.^10^ The microarray dataset is available from Gene Expression Omnibus (GSE21545).

To analyze the gene expression of the IL6 receptors in carotid atherosclerotic plaques, we designed and validated oligoprimers to selectively amplify the membrane-bound (*IL6R* and *GP130*) and soluble (*sIL6R* and isoform s*GP130-RAPS* [s*GP130*]) IL6 receptors. The experimental design allowed the co-amplification in two separate reactions: *IL6R*/*sIL6R* and *GP130/*s*GP130*. In each reaction, two housekeeping genes, *β-ACTIN* and *GADPH*, were used to normalize differences in gene expression. The same batch of reagents was used in each amplification reaction. A detailed description of the experimental protocol is reported in the online Supplemental Material.

In semi-qRT-PCR, the cycle threshold (*C*_T_) for a specific gene is a quantification of the amount of copies in the sample. The difference between the *C*_T_ for each target gene and the average *C*_T_ of the two housekeeping genes *β-ACTIN* and *GADPH* (∆*C*_T_) was calculated for every sample. Comparison of gene expression levels were based on ∆*C*_T_ values.^16^ Since gene expression is inversely related to the ∆*C*_T_ threshold value, the negative ∆*C*_T_ value was used in the analyses to facilitate interpretation of the results.

### Statistics

All continuous variables are presented as median with an IQR, while binary variables are presented as numbers (percentages). The significance level was set to 5%.

In the main analysis, the relative expression of *IL6, IL6R, sIL6R, GP130*, and *sGP130* in carotid atherosclerotic plaques was examined. The correlation between plasma and plaque *IL6* and IL6 receptors gene expression levels was tested by Spearman’s rank correlation test. In exploratory analyses, the expression patterns were analyzed in symptomatic and asymptomatic patients and in statin-treated and non-treated patients. Differences in plaque gene expression and plasma levels between subgroups of study participants and in relation to time (⩽ 30 vs > 30 days) from event to surgery in symptomatic patients were analyzed using the Kruskal–Wallis rank test.

All statistical analyses were performed in Stata statistical software, Release 14 (StataCorp LP, College Station, TX, USA).

## Results

Table 1 summarizes the clinical characteristics of the study participants. The majority of patients were male and the median age was 73 years (IQR 65–78). Nearly 68% of the study population had suffered an ischemic cerebrovascular event prior to surgery (i.e. were categorized as symptomatic). Among these participants, the median time from event to surgery was 36 days. Among the cardiovascular risk factors, hypertension was the most prevalent and the absolute majority of patients were on statin treatment.

**Table 1.** Clinical characteristics of the study population (*n* = 78).

Age, years	73 (65–78)
Male sex (%)	58 (74.4)
Stroke/TIA (%)	53 (67.9)
Time to surgery, days	36.5 (16–77)
Hypertension (%)	67 (85.9)
Diabetes mellitus (%)	23 (29.5)
BMI, kg/m^2^	26.0 (23.7–28.6)
Statin treatment (%)	65 (85.5)
Current smoking (%)	18 (26.5)

Continuous variables (age, BMI, time to surgery, plasma IL6, sIL6R, sgp130, and hsCRP levels) are expressed as median (IQR). Binary variables (sex, history of hypertension, diabetes mellitus, smoking, and treatment with statins) are expressed as numbers (%).

Stroke/TIA displays the number of patients classified as symptomatic with an ischemic stroke or TIA at the time of inclusion. Missing data: time to event (*n* = 3), statin treatment (*n* = 2), BMI (*n* = 6), and smoking (*n* = 10).

BMI, body mass index; hsCRP, high-sensitivity C-reactive protein; IL6, interleukin 6; sgp130, soluble glycoprotein 130; sIL6R, soluble IL6 receptor; TIA, transient ischemic attack.

### IL6 receptor gene expression in carotid artery plaques

**Figure 2.**
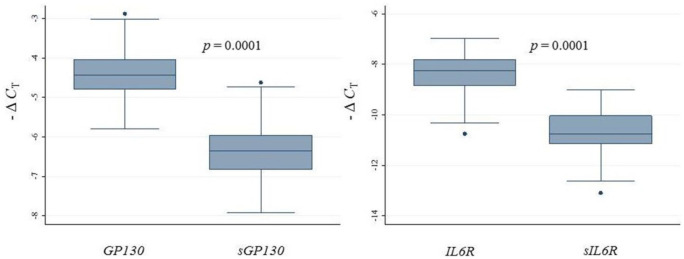
Relative gene expression levels of *GP130, sGP130, IL6R*, and *sIL6R* in carotid artery plaques in all subjects included (*n* = 78) measured by semi-qRT-PCR and reported as – Δ*C*_T_. Kruskal–Wallis was used to analyze the relative differences in expression between receptors. – Δ*C*_T_, negative delta cycle threshold; Semi-qRT-PCR, semi-quantitative real-time polymerase chain reaction.

*IL6* expression in carotid atherosclerotic plaques has already been reported.^10^ We found that *IL6R, sIL6R, GP130*, and *sGP130* were also expressed in carotid plaques. Figure 2 shows that the relative gene expression levels of both membrane-bound receptors (*IL6R* and *GP130*) were significantly higher than their respective soluble isoform (*sIL6R* and *sGP130)*.

### Circulating inflammatory biomarkers

Plasma levels of the components of the IL6 signaling pathway and high-sensitivity CRP (hsCRP) are presented in the upper panel of Table 2.

**Table 2.** Circulating plasma levels of IL6, sIL6R, sgp130, and hsCRP.

	All (*n* = 78)	Symptomatic (*n* = 53)	Asymptomatic (*n* = 25)	*p*
IL6	2.5 (1.4–3.3)	2.5 (1.2–3.3)	2.3 (1.4–3.8)	0.74
sIL6R	66.3 (50.3–82.7)	66.1 (50.4–84.3)	67.0 (46.0–79.7)	0.63
sgp130	373.9 (339.5–421.2)	369.1 (334.5–410.4)	386.5 (345.8–427.8)	0.21
hsCRP	2.75 (1.3–5.0)	2.8 (1.3–6.2)	2.9 (1.1–4.4)	0.64
		Statin (*n* = 65)	No statin (*n* = 11)	*p*
IL6		2.5 (1.5–3.3)	1.5 (0.9–4.4)	0.19
sIL6R		67.0 (51.2–83.4)	60.9 (35.4–70.0)	0.05
sgp130		373.2 (339.5–413.0)	395.3 (319.1–448.9)	0.38
hsCRP		2.8 (1.3–4.5)	3.2 (1.7–7.1)	0.54

Data presented as median (IQR).

Plasma concentrations expressed in pg/mL for IL6, ng/mL for sIL6R and sgp130, and mg/L for hsCRP.

Kruskal–Wallis was used to calculate the *p*-value for differences between subgroups. Missing information on IL6 (*n* = 9) and hsCRP (*n* = 12).

hsCRP, high-sensitivity C-reactive protein; IL6, interleukin 6; sgp130, soluble glycoprotein 130; sIL6R, soluble IL6 receptor.

### Correlation between IL6 and IL6 receptor gene expression in plaques and IL6, sIL6R, and sgp130 circulating levels

**Figure 3.**
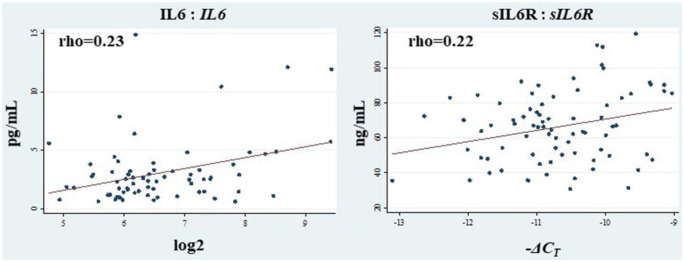
Correlations between plasma levels of IL6 and sIL6R and the expression of their corresponding genes in carotid plaques. Correlations between plasma levels of IL6 (pg/mL) and sIL6R (ng/mL) and carotid plaque gene expression of *IL6* (log2) and *sIL6R* (– Δ*C*_T_) were estimated by Spearman’s rank correlation coefficient (rho) describing the level and direction of the correlation. IL6, interleukin 6; sIL6R, soluble IL6 receptor.

*IL6* and *sIL6R* displayed a moderate, borderline significant, positive correlation (both *p* = 0.05) with IL6 and sIL6R plasma levels, graphically presented in Figure 3. No additional correlation was observed between gene expression in plaques and plasma levels, as reported in online Supplemental Table 3.

### Exploratory analyses of the IL6 signaling pathway gene expression and plasma levels in clinically relevant subgroups of patients

#### Symptomatic versus asymptomatic patients

Notwithstanding that all the patients included in the present study suffer from a severe atherosclerotic disease, we wanted to explore potential differences in the expression and circulating levels of these biomarkers in patients who had already suffered a stroke/TIA as compared to those who had not. We compared plasma and gene expression levels in symptomatic (*n* = 53) and asymptomatic (*n* = 25) patients. According to the definition used in the BiKE study, patients were classified as symptomatic if they had suffered a cerebrovascular event before undergoing surgery.

No differences were observed in plasma levels of IL6 and its soluble receptors between symptomatic and asymptomatic patients (Table 2, upper section). In the group of symptomatic patients, plasma levels of sIL6R were higher in those where ⩽ 30 days had passed from the cerebrovascular event to surgery as compared to subjects with a longer delay (Table 3). No differences in gene expression were observed in relation to time from event to surgery (data not shown).

**Table 3.** IL6, sIL6R, and sgp130 levels stratified by time from event to CEA.

	⩽ 30 days	> 30 days	*p*
IL6	2.5 (1.2–4.8)	2.7 (1.4–3.2)	0.48
sIL6R	73.3 (64.4–90.0)	55.8 (48.2–74.7)	0.02
sgp130	368.7 (340.6–413.0)	371.1 (312.9–400.9)	0.43
hsCRP	2.8 (1.5–6.4)	2.2 (1.2–6.2)	0.71

Median (IQR) plasma levels of IL6 (pg/mL), sIL6R and sgp130 (ng/mL), and hsCRP mg/L according to time from cerebrovascular event to CEA ⩽ and > 30 days.

The *p*-value is calculated by Kruskal–Wallis. Missing information on time in IL6 (*n* = 10), sIL6R, and sgp130 (*n* = 3) and hsCRP (*n* = 14).

CEA, carotid endarterectomy; hsCRP, high-sensitivity C-reactive protein; IL6, interleukin 6; sgp130, soluble glycoprotein 130; sIL6R, soluble IL6 receptor.

**Figure 4.**
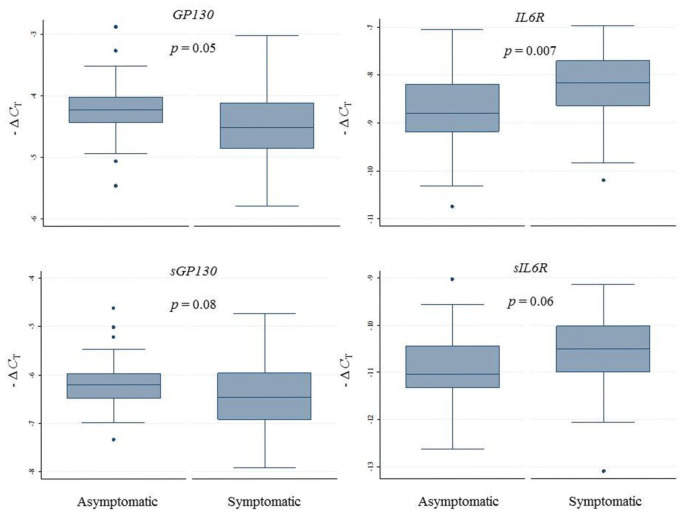
Relative gene expression levels of the membrane-bound and soluble IL6 receptors in carotid artery plaques of asymptomatic and symptomatic patients. Relative gene expression levels quantified by semi-qRT-PCR and expressed as – Δ*C*_T_. The *p*-value is for differences between asymptomatic and symptomatic subjects. – Δ*C*_T_, negative delta cycle threshold; IL6, interleukin 6; semi-qRT-PCR, semi-quantitative real-time polymerase chain reaction.

*IL6R* expression levels were higher in symptomatic as compared to asymptomatic patients. Similarly, *sIL6R* expression was slightly higher in symptomatic subjects, albeit the difference did not attain statistical significance, as summarized in Figure 4, and reported in the online Supplemental Table 4. No difference in *IL6* expression was observed (data not shown).

#### Statin-treated versus non-statin-treated

Statins are widely used in primary and secondary prevention of atherosclerotic-related diseases and are known to exert an anti-inflammatory effect.

In statin-treated patients, plasma sIL6R were higher than in non-treated patients (Table 2, lower section). No differences were observed for any of the other plasma inflammatory biomarkers.

**Figure 5.**
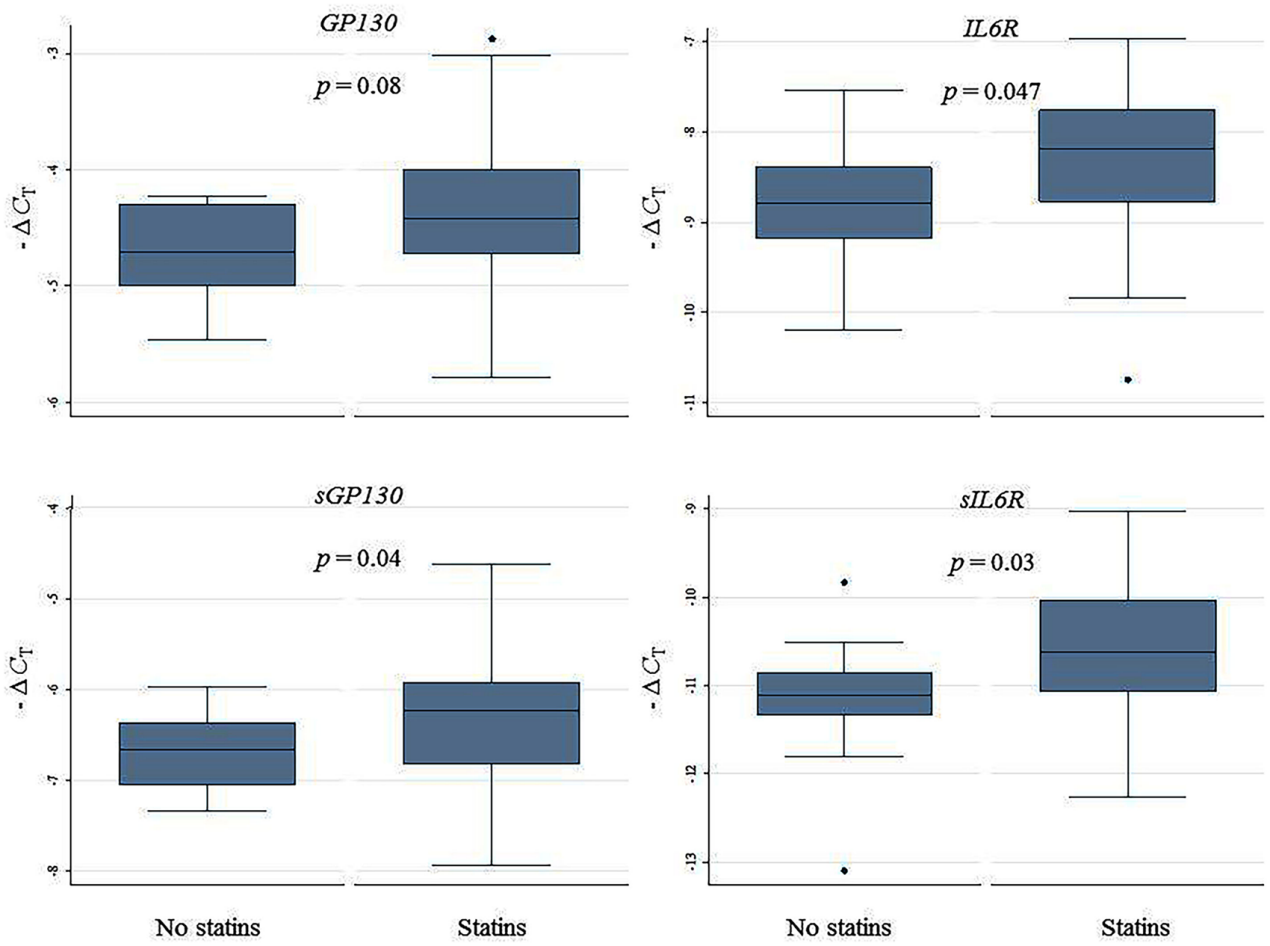
Relative gene expression of the membrane-bound and soluble IL6 receptors in carotid artery plaques of statin-treated and non-treated patients. Relative gene expression levels quantified by semi-qRT-PCR and expressed as – Δ*C*_T_. The *p*-value is for differences between statin-treated (*n* = 65) and non-treated (*n* = 11) patients. Missing information on lipid-lowering therapy (*n* = 1) and monotherapy with ezetimibe (*n* = 1) not included in the present analysis. IL6, interleukin 6; – ΔC_T_, negative delta cycle threshold; semi-qRT-PCR, semi-quantitative real-time polymerase chain reaction.

As shown in Figure 5 and online Supplemental Table 4, statin-treated patients (*n* = 65) exhibited relatively higher *IL6R, sIL6R*, and *sGP130* expression in plaques as compared to non-treated patients (*n* = 11). No difference in *IL6* expression was observed (data not shown).

## Discussion

We demonstrate for the first time that all components of the IL6 classical and trans-signaling pathways are expressed in high-grade carotid atherosclerotic plaques. In addition, our findings suggest that plasma levels of the components in the active IL6 trans-signaling complex (IL6:sIL6R) correlate moderately with the corresponding genes in carotid plaques. These results generate a novel hypothesis on a potential cross-talk between systemic and local IL6 trans-signaling in carotid atherosclerotic disease.

We have investigated the presence of IL6 signaling in advanced manifest atherosclerosis in BiKE, a Swedish cohort of patients in Stockholm undergoing CEA due to carotid artery stenosis. Given that carotid artery stenosis is a well-established risk factor for ischemic stroke, finding biomarkers indicating presence of carotid atherosclerotic plaques is of essence in preventing future cerebrovascular events.^17^ Hence, unravelling the role of IL6 signaling may have important clinical implications for prevention and treatment of atherosclerosis.

In line with previous studies,^18,19^ we found that *IL6* was expressed in carotid atherosclerotic plaques, but no difference in *IL6* expression was observed between symptomatic and asymptomatic patients. Circulating IL6 levels have been shown to be higher in patients with carotid plaques compared to those without but, consistent with our findings, no difference in IL6 circulating levels have been demonstrated in symptomatic versus asymptomatic patients with carotid atherosclerotic plaques.^3^ The absence of differences in *IL6* expression and IL6 circulating levels between symptomatic and asymptomatic patients with large vessel disease is controversial^3,20,21^ and can possibly be explained by the time elapse between cerebrovascular symptoms and surgery. We did not, however, observe any difference in either the expression or IL6 circulating levels over time. IL6 circulating levels in our study possibly mirror the longstanding low-grade inflammation secondary to severe atherosclerotic disease. Plasma sIL6R levels, on the other hand, decreased with time from the cerebrovascular event to surgery in agreement with the increased levels seen in acute myocardial infarction.^22,23^ The presence of an autocrine/paracrine IL6 trans-signaling in the vessel wall has been suggested in in vitro studies, and on human vascular smooth muscle cells it has been demonstrated that locally secreted IL6 and sIL6R, shed from myeloid cells, upregulate gp130 receptor expression, thus enabling trans-signaling.^24^ More recently, studies on human endothelial cells showed that IL6 classical and trans-signaling activate specific intra-cellular signaling cascades.^25^ Moreover, the balance between the two IL6 signaling pathways has been analyzed using a computational approach and proposing that, on hepatocytes, IL6 trans-signaling is able to override classical signaling when the protein expression of gp130 exceeds that of IL6R.^26^ Our study design does not allow us to calculate the *GP130/IL6R* gene expression ratio in carotid atherosclerotic plaques. However, the two membrane-bound receptors were highly expressed in carotid plaques, thus suggesting that both signaling pathways may be active in advanced atherosclerotic plaque. Of interest, plasma sIL6R correlated moderately with *sIL6R* plaque expression. These results may generate novel hypotheses on the existence of a cross-talk between circulating and local IL6 trans-signaling.

In this small subset of the BiKE study, the majority of patients had experienced a cerebrovascular event in the last 6 months prior to surgery. In these symptomatic patients, there was a relatively higher plaque expression of *IL6R* as compared to asymptomatic patients. This is in line with earlier results from the BiKE cohort demonstrating that expression of genes associated with inflammation were upregulated in carotid atherosclerotic plaques obtained from symptomatic patients as compared with those obtained from asymptomatic ones.^15^

The majority of study participants were treated with statins. The soluble trans-signaling receptor genes *sIL6R* and *sGP130* were overexpressed in plaques of patients on statins as compared to non-treated patients. When investigating differences in plasma levels of the IL6 trans-signaling receptors, only sIL6R differed with higher levels in statin-treated patients. Low cholesterol levels have been demonstrated to induce IL6R shedding, thus increasing circulating levels of sIL6R,^27^ while statin treatment at the same time is known to exert anti-inflammatory effects on carotid atherosclerotic plaques with decreasing circulating levels of IL6 and CRP and signs of more stable plaques.^15,28,29^ The observation of a possible upregulation of IL6 trans-signaling in the presence of statin treatment indirectly confirms our previous findings where IL6 trans-signaling was a strong predictor of CVE in the presence of low-normal cholesterol levels and further supports the hypothesis that modulation of inflammation in atherosclerosis may represent a novel treatment moiety in selected groups of patients.

Taken together, these findings suggest that IL6 plays an important and possibly differential role in physiological and pathological mechanisms in the vascular wall and that IL6 trans-signaling may act as an autocrine/paracrine signaling system in high-grade carotid plaques. In a future perspective, dampening IL6-driven inflammation in the vessel wall with recombinant sgp130Fc could possibly be used to prevent ischemic stroke in patients at high risk of large vessel atherosclerosis. Hence, finding inflammatory biomarkers mirroring the atherosclerotic burden is essential to identify patients amenable to treatment and to monitor treatment effects. In particular, the role of sIL6R as a marker of carotid atherosclerosis merits further investigation. We have observed higher levels of sIL6R in statin-treated patients. Although this finding is hard to interpret in light of the known anti-inflammatory role of statins, we can only speculate that sIL6R might drive an inflammatory reaction independent from the inflammatory response dampened by statins and/or that, in the presence of advanced atherosclerotic lesions, anti-inflammatory drugs are needed on top of the standard treatment to resolve a chronic long-standing inflammatory reaction.

### Strengths and limitations

This is the first study analyzing IL6 signaling in carotid atherosclerotic plaques and the first to present plasma sIL6R as a potential new marker of inflammatory activity in carotid plaques. In addition, the BiKE study biobank is a unique population-based real-life cohort designed to explore mechanisms of atherosclerosis.

The major limitation of this study is the relatively small sample size of the population studied. Moreover, all analyses are cross-sectional (i.e. temporality and causality cannot be demonstrated with this study design) and thus larger longitudinal cohort studies are needed to assess the differential role of IL6 classical and IL6 trans-signaling in atherosclerotic plaque development. In order to assess tissue-specific expression, we have not analyzed local protein levels in carotid atherosclerotic plaques, which would have been of value to do as sIL6R is mainly produced by proteolytic cleavage. On the other hand, sgp130 is primarily produced through alternative splicing and exist in three isoforms, albeit we quantified solely one, sgp130-RAPS, as it is the only sgp130 isoform verified by Western blot.^30^ Sgp130-RAPS was originally detected in patients with rheumatoid arthritis, hence there is a risk of bias in patients with this condition, although it is relatively rare and to our knowledge none of the patients included in this study had clinically overt rheumatic disease. Furthermore, the receptors were co-amplified in pairs (*IL6R; sIL6R* and *GP130; sGP130*), thus the differences in expression levels between the membrane-bound and soluble *IL6R* and *GP130* could not be analyzed. In addition, since the plaques were halved during surgery and only one half was prepared for RNA analysis, there is a risk of under or overestimate receptor expression if their expression level is unevenly distributed in the plaque. Moreover, the study was restricted to analyses of late stage lesions; consequently, we cannot draw any conclusions on the expression levels of these genes in early atherosclerotic lesions. It is also well known that there is a substantial diurnal variation of circulating IL6.^31^ In the present study, this variation could potentially explain the relatively low correlation between plasma and tissue expression levels. Finally, owing to the exploratory nature of the subgroup analyses, and with the lack of formal statistical significance, firm conclusions on the differences in gene expression and plasma levels between subgroups cannot be drawn, albeit the results merit further investigation into IL6 signaling in arterial vasculature.

## Conclusion

*IL6* and the membrane-bound and soluble *IL6R* and *GP130* receptor genes are all expressed in carotid atherosclerotic plaques, and *sIL6R* expression in atherosclerotic plaques correlates with sIL6R plasma levels.

## Supplemental Material

sj-docx-1-vmj-10.1177_1358863X20977662 – Supplemental material for Expression of Interleukin 6 signaling receptors in carotid atherosclerosis Click here for additional data file. Supplemental material, sj-docx-1-vmj-10.1177_1358863X20977662 for Expression of Interleukin 6 signaling receptors in carotid atherosclerosis by Louise Ziegler, Jasmin Lundqvist, Kristian Dreij, Håkan Wallén, Ulf de Faire, Gabrielle Paulsson-Berne, Ulf Hedin, Ljubica Matic and Bruna Gigante in Vascular Medicine
