# Supplementary material for: Expression of Interleukin 6 signaling receptors in carotid atherosclerosis
Source: Vasc Med. 2020 Dec 22;26(1):3–10. doi: 10.1177/1358863X20977662 (PMC7879223; doi:10.1177/1358863X20977662)
Supplement: sj-docx-1-vmj-10.1177_1358863X20977662 – Supplemental material for Expression of Interleukin 6 signaling receptors in carotid atherosclerosis [file sj-docx-1-vmj-10.1177_1358863X20977662.docx]

# SUPPLEMENTAL MATERIAL

# Expression of IL6 signaling receptors in carotid atherosclerosis

Louise Ziegler, MD, PhD ^1,2^; Jasmin Lundqvist ^3^; Kristian Dreij, PhD ^4^; Håkan Wallén, MD, PhD ^1,5^; Ulf de Faire, MD, PhD ^6,7^; Gabrielle Paulsson-Berne, PhD ^3^; Ulf Hedin, MD, PhD ^3,8^; Ljubica Matic, PhD ^9^; Bruna Gigante MD, PhD ^3,5^.

**Departments and institutions:** ^1^ Department of Clinical Sciences, Danderyd Hospital, Karolinska Institutet; ^2^ Department of Medicine, Danderyd Hospital; ^3^ Cardiovascular Medicine Unit, Department of Medicine, Karolinska Institutet; ^4^ Unit of Biochemical Toxicology, Institute of Environmental Medicine, Karolinska Institutet; ^5^ Department of Cardiology, Danderyd Hospital; ^6^ Unit of Cardiovascular and Nutritional Epidemiology, Institute of Environmental Medicine, Karolinska Institutet; ^7^ Department of Cardiology, Karolinska University Hospital; ^8^ Department of Vascular Surgery, Karolinska University Hospital and ^9^ Department of Molecular Medicine and Surgery, Karolinska Institutet, Stockholm, Sweden

**Corresponding author contact information:**

Louise Ziegler, MD, PhD

Department of Clinical Sciences Danderyd Hospital

Karolinska Institutet

S-182 57 Stockholm, Sweden

Tel: +46 8 123 580 48/550 00

Fax: +46 8 524 800 00

E-mail: [louise.dencker-ziegler@sll.se](mailto:louise.dencker-ziegler@sll.se)

**Content:**

Supplemental Methods

Supplemental Figure 1-2

Supplemental Table 1-4

Supplemental References

## Supplemental Methods

### Design of oligoprimers to amplify *IL6R*, s*IL6R*, *GP130*, s*GP130*-*RAPS* and the two housekeeping genes *β-ACTIN* and *GAPDH*

Oligoprimers to amplify *IL6R, sIL6R, GP130,* s*GP130-RAPS* (s*GP130*) and the two housekeeping genes *β-ACTIN* and glyceraldehyde 3-phosphate dehydrogenase (*GAPDH)* were designed from sequences reported in GenBank.

Supplemental Table 1 show the oligoprimer sequences designed for amplification of the two housekeeping genes used to normalize gene expression in the present study: *β-ACTIN* and *GADPH*. *β-ACTIN* oligoprimers were designed on cDNA sequence NM_001101 and *GADPH* primers on cDNA sequence NM_002046.

Oligoprimers for *IL6R, sIL6R, GP130* and s*GP130-RAPS* were designed to selectively amplify each receptor.

Supplemental Figure 1 shows a schematic representation of the *IL6R* cDNA sequence NM_000565.4 (base pairs [bp] 1261-1560) along with the oligoprimers designed to amplify the two IL6 receptors, *IL6R* and s*IL6R*. As a result of alternative splicing, s*IL6R* lacks a 94-bp fragment which includes the entire *IL6R* transmembrane (TM) region, underlined in the figure. The oligoprimers to amplify s*IL6R* are reported in bold black in the figure: the reverse primer spans across the splicing junction. On the contrary, to amplify the membrane bound *IL6R* the reverse primer (in red) was designed in the TM region.

Supplemental Figure 2 shows a schematic representation of *GP130* cDNA sequences. Panel A shows the nucleotide sequence of the *GP130* isoform 1 corresponding to the membrane bound receptor (GenBank accession number: NM_002184.3) and panel B the *GP130* isoform 2 (NM_175767.3) sequence also known as s*GP130-RAPS* (1-3). The circulating sgp130-RAPS variant differs from the full length sgp130 since it produced by alternative splicing of exon 9 (1). The splicing creates a stop codon and results in a shorter protein that lacks the TM domain (exon 15) (1). Among the described sgp130 isoforms, only sgp130-RAPS has been verified by Western blot (4) and is the most widely expressed (5).

### Validation of oligoprimers designed to amplify *IL6R*, s*IL6R*, *GP130,* s*GP130-RAPS* and the two housekeeping genes *β-ACTIN* and *GAPDH*

To validate the primer sequences, preliminary experiments were performed on cDNA generated through reverse transcription with the QuantiTect Reverse Transcription Kit (QIAGEN GmbH, Hilden, Germany) and random primers from total RNA extracted from HepG2 cells using standard protocols.

Traditional PCR was performed for each primer set with 0.5 pmol/μl of forward and reverse primers, 0.02 U/μl Phusion DNA polymerase, 1X Phusion HF buffer, 200 pmol/μl dNTP, 0.5 ng/μl template cDNA in a 20 μl reaction. The cycles used were 98 °C for 2 min, followed by 35 cycles at 98 °C for 10 s and 60 °C for 30 s and 72 °C for 30 s. The PCR products were then loaded on a 3% agarose gel with GelRed® Nucleic Acid Gel Stain (Biotium, Fremont, CA, USA). After gel electrophoresis and UV-examination, fragments were cut out and purified using the QIAquick Gel Extraction Kit (QIAGEN GmbH, Hilden, Germany). Amplification of correct PCR products was verified through sequencing at the KIGene core facility (Karolinska Institutet, Sweden).

### Probe design

TaqMan^®^ probes were custom designed by Applied Biosystems (Thermo Fisher Scientific) to be compatible and selective for the oligoprimer/gene sequences.

IL6 signaling receptors were made as MGB probes and housekeeping genes as QSY probes, so that two MGB probes could be co-amplified with the two housekeeping genes in one multiplex. Applied Biosystems manufactured a designed assay mix (containing primers and probes) for each IL6 receptor and for *β-ACTIN* and *GAPDH* (Supplemental Table 2). Applied Biosystems vouches that each assay has passed bioinformatic quality control and is predicted to be specific. Lastly, the assays are not predicted to interact with each other in a multiplex reaction.

As last step in the validation of the primers and probe sequence, the cDNA obtained from the HEP2G cells was amplified at different concentration (0.2-0.5 ng/microliter) in singleplex and multiplex reaction using in all assay combinations. From these experiments, an optimal oligoprimer/probe concentration was derived. In the multiplex reaction, *IL6R, sIL6R, GP130* and s*GP130-RAPS* were used at a concentration of 900 nanomoles/L (nmol/L) for each primer and mixed with 250 nmol/L of probe while *β-ACTIN* and *GAPDH* were used at a concentration 225 nmol/L for each primer and mixed with 62.5 nmol/L probe.

### Real time semi-quantitative PCR

Total RNA (3.3 ng/ul) was extracted from carotid endarterectomies and reverse transcribed to cDNA using the QuantiTect Reverse Transcription Kit (QIAGEN GmbH, Hilden, Germany) and random primers as described previously (6).

Five nanograms of cDNA was used for each semi-qRT-PCR reaction, which were run in duplicates, using the TaqMan Multiplex Master Mix on an Applied Biosystems 7500 Real-Time PCR system. The cycles used were 95°C for 20 s, followed by 40 cycles at 95°C for 3 s and 60°C for 30 s. The genes were multiplexed in two sets. The first included *IL6R, sIL6R, β-ACTIN* and *GAPDH*, and the second *GP130,* s*GP130-RAPS, β-ACTIN* and *GAPDH*. Oligoprimers/probe concentration in each reaction are outlined above.

JL and KD, who run the semi-qRT-PCR, were not aware of the symptomatic and asymptomatic status of the patients included in the study. *IL6R, sIL6R, GP130* and s*GP130*-RAPS expression was quantified by the comparative cycle threshold (*C*_T_) method (7). ∆*C*_T_ is the difference between the *C*_T_ of the target gene and the average *C*_T_ for *β-ACTIN* and *GAPDH*.

## *Supplemental Figures*

Supplemental Figure 1. Schematic representation of the *IL6R* cDNA sequence NM_000565 (base pairs [bp] 1261-1560) along with the TM domain and oligoprimer sequences chosen to amplify *IL6R* and the *sIL6R*.

1261 ACGAGGTGTC CACC**CCCATG CAGGCACTTA CTACT**AATAA AGACGATGAT AATATTCTCT

1321 TCAGAGATTC TGCAAATGCG ACAAGCC**TCC** **CAG**TGCAAGA TTCTTCTTCA GTACCACTGC

1381 CCACATTCCT GGTTGCTGGA GGGAGCCTGG CCTTCGGAAC GCTCCTCTGC ATTGCCATTG

1441 TTCTGAG***GTT*** ***CAAGA*AGACG** **T**GGAAGCTGC GGGCTCTGAA GGAAGGCAAG ACAAGCATGC

1501 ATCCGCCGTA CTCTTTGGGG CAGCTGGTCC CGGAGAGGCC TCGACCCACC CCAGTGCTTG

|  | **Oligoprimer sequences** | **Amplicon length (bp)** |
| --- | --- | --- |
| *IL6R forward* | 5’-AGAGATTCTGCAAATGCGACAAG-3’ | 133 |
| *IL6R* reverse | 5’-TCTTGAACCTCAGAACAATGGC-3’ |  |
| *sIL6R* forward | 5’-CCCATGCAGGCACTTACTACT-3’ | 93 |
| *sIL6R* reverse | 5’-ACGTCTTCTTGAACCTGGGA-3’ |  |

In red, the forward (bp 1323-1345) and reverse oligoprimers (bp 1434-1455) used to amplify the membrane-bound *IL6R*. The reverse oligoprimer lies in the 94 bp TM domain (underlined)

In bold black, the forward (bp 1274-1294) and reverse primer (bp 1348-1353 and 1448-1461) used to amplify the *sIL6R*. The reverse primer crosses the splicing junction and overlaps from bp 1448 to bp 1455 with the *IL6R* reverse primer (highlighted in bold cursive red).

Oligoprimer sequences and amplicon length are reported in the table under the figure.

### **Supplemental Figure 2.** Panel A. Schematic representation of the *GP130* cDNA sequence NM_002184.4 (bp 1251-1420) and the oligoprimers chosen to amplify the gene coding membrane-bound gp130. Panel B. Schematic representation of the s*GP130-RAPS* cDNA sequence NM_175767.3 (bp 1201-1420) and the oligoprimers chosen to amplify the isoform coding sgp130-RAPS.

**PANEL A.**

1251 AAGATAGACC ATCTAAAGCA CCAAGTTTCT GGTATAAAAT **GATCCATCC CATACTCAAG**

1311 **G**CTACAGAAC TGTACAACTC GTGTGGAAGA CATTGCCTCC TTTTGAAGCC AATGGAAAAA

1371 TCTTGGATTA TGAA**GTGACT CTCACAAGAT GGA**AATCACA TTTACAAAAT TACACAGTTA

**PANEL B.**

1231AGCAAGTGGG ATCACCTATG AAG**ATAACAT TGCCTCCTTT TGAAGCC**AAT GGAAAAATCT

1291 TGGATTATGA AGTGACTCTC ACAAGATGGA AATCACATTT ACAAAATTAC ACAGTTAATG

1351 CCACAAAACT GACAGTAAAT **CTCACAAATG ATCGCTATCT AGC**AACCCTA

|  | Oligoprimer sequences | Amplicon length (bp) |
| --- | --- | --- |
| *GP130* forward | 5’-GATCCATCCCATACTCAAGG-3’ | 112 |
| *GP130* reverse | 5’-TCCATCTTGTGAGAGTCAC-3’ |  |
| s*GP130* forward | 5’-ATAACATTGCCTCCTTTTGAAGCC-3’ | 140 |
| s*GP130* reverse | 5’-GCTAGATAGCGATCATTTGTGAG-3’ |  |

**Panel A**. In bold black the forward (bp 1291-1311) and reverse oligoprimer (bp 1384-1403) selected to amplify the membrane-bound *GP130*. Forward oligoprimer lies in the sequence coding exon 9 (underlined) with the donor and acceptor sequences at the splicing junction underlined in red. Alternative splicing of exon 9 results in the alternative *GP130* transcript (reported in Panel B) which translates in the soluble isoform sgp130-RAPS (s*GP130*).

**Panel B**. In bold black the forward (bp 1254-1276) and reverse (1371-1393) oligoprimers selected to amplify the soluble gp130-RAPS (s*GP130*). As compared to the membrane bound gp130, this isoform lacks exon 9. The forward oligoprimer spans the splicing junction, underlined in red. The alternative splicing creates a stop codon “TAA” which results in the translation of the shorter circulating isoform sgp130-RAPS.

Oligoprimer sequences and amplicon length are reported in the table under the figure.

## *Supplemental Tables*

### Supplemental **Table 1.** Oligonucleotide primers designed to amplify housekeeping genes, *β-ACTIN* and *GAPDH*.

| Gene | Primer | Sequence | Amplicon length (bp) |
| --- | --- | --- | --- |
| *β-ACTIN* | Forward | 5’-GTGATGGACTCCGGTGACG-3′ | 191 |
|  | Reverse | 5′-TTCTCCTTAATGTCACGCACGAT-3′ |  |
| *GAPDH* | Forward | 5′-ACCCACTCCTCCACCTTTGAC-3′ | 100 |
|  | Reverse | 5′-TGTTGCTGTAGCCAAATTCGTT-3′ |  |

### **Supplemental Table 2.** ID for TaqMan® probes generated at Applied Biosystem

| **Name of gene** | **Probe ID** |
| --- | --- |
| *IL6R* | AI20VIM |
| *sIL6R* | AI39TOU |
| *GP130* | AI5IRU2 |
| s*GP130-RAPS* | AI6RP1A |
| *β-ACTIN* | ACTIN_nD.QSY |
| *GAPDH* | GAPDA_nD.QSY |

### Supplemental Table 3. Correlation between plasma levels and expression in plaques

| Plaque expression | **Plasma concentration** | | |
| --- | --- | --- | --- |
|  | **IL6** | **sIL6R** | **sgp130** |
| ***IL6*** | 0.23 (0.05) | 0.08 (0.51) | 0.08 (0.49) |
| ***IL6R*** | -0.04 (0.74) | 0.09 (0.44) | -0.07 (0.52) |
| ***sIL6R*** | 0.08 (0.50) | 0.22 (0.05) | 0.01 (0.97) |
| ***GP130*** | 0.11 (0.38) | 0.18 (0.12) | -0.10 (0.38) |
| ***sGP130*** | 0.14 (0.25) | 0.19 (0.09) | -0.12 (0.28) |

**Supplemental Table 3.** Spearman correlation coefficient, rho (p-value) for circulating IL6 and receptors in plasma and expression in plaques. Plaque expression estimated by - Δ*C*_T_ except for *IL6* expression estimated by log2.

### Supplemental Table 4. Levels of *GP130*, *sGP130*, *IL6R* and *sIL6R* expression in asymptomatic and symptomatic patients (upper panel) and in non-statin-treated and statin-treated patients (lower panel).

| **Gene** | **Subgroup** | **25^th^ perc** | **50^th^ perc** | **75^th^ perc** |
| --- | --- | --- | --- | --- |
| ***GP130*** | Asymptomatic | -4.43 | -4.23 | -4.01 |
|  | Symptomatic | -4.86 | -4.52 | -4.12 |
| ***sGP130*** | Asymptomatic | -6.48 | -6.20 | -5.98 |
|  | Symptomatic | -6.93 | -6.46 | -5.96 |
| ***IL6R*** | Asymptomatic | -9.19 | -8.80 | -8.19 |
|  | Symptomatic | -8.65 | -8.16 | -7.71 |
| ***sIL6R*** | Asymptomatic | -11.34 | -11.05 | -10.44 |
|  | Symptomatic | -11.00 | -10.51 | -10.02 |
| ***GP130*** | No statin | -4.99 | -4.71 | -4.30 |
|  | Statin | -4.72 | -4.42 | -4.00 |
| ***sGP130*** | No statin | -7.04 | -6.66 | -6.36 |
|  | Statin | -6.81 | -6.23 | -5.92 |
| ***IL6R*** | No statin | -9.19 | -8.79 | -8.40 |
|  | Statin | -8.77 | -8.19 | -7.76 |
| ***sIL6R*** | No statin | -11.34 | -11.12 | -10.85 |
|  | Statin | -11.06 | -10.63 | -10.04 |

**Supplemental** **Table 4.** Gene expression quantified by real time semi-quantitative PCR, expressed as negative Δ*C*_T_ and presented as the 50^th^ percentile (perc) and the interquartile range, i.e. 25^th^ – 75^th^ perc, in sub groups defined by symptomatic or asymptomatic carotid artery stenosis and statin treatment or not in the BiKE cohort.

## *References*

1. Diamant M, Rieneck K, Mechti N et al. Cloning and expression of an alternatively spliced mRNA encoding a soluble form of the human interleukin-6 signal transducer gp130. FEBS letters 1997;412:379-84.

2. Szalai C, Toth S, Falus A. Exon-intron organization of the human gp130 gene. Gene 2000;243:161-6.

3. Tanaka M, Kishimura M, Ozaki S et al. Cloning of novel soluble gp130 and detection of its neutralizing autoantibodies in rheumatoid arthritis. The Journal of clinical investigation 2000;106:137-44.

4. Sommer J, Garbers C, Wolf J et al. Alternative intronic polyadenylation generates the interleukin-6 trans-signaling inhibitor sgp130-E10. J Biol Chem 2014;289:22140-50.

5. Wolf J, Waetzig GH, Chalaris A et al. Different Soluble Forms of the Interleukin-6 Family Signal Transducer gp130 Fine-tune the Blockade of Interleukin-6 Trans-signaling. J Biol Chem 2016;291:16186-96.

6. Razuvaev A, Ekstrand J, Folkersen L et al. Correlations between clinical variables and gene-expression profiles in carotid plaque instability. European journal of vascular and endovascular surgery : the official journal of the European Society for Vascular Surgery 2011;42:722-30.

7. Schmittgen TD, Livak KJ. Analyzing real-time PCR data by the comparative C(T) method. Nature protocols 2008;3:1101-8.
